# 1 Burn Center Verification and Safety-net Status. Are There Differences in Discharge to Inpatient Rehabilitation?

**DOI:** 10.1093/jbcr/irae036.001

**Published:** 2024-04-17

**Authors:** Devi Lakhlani, John Kevin Bailey, Samantha Steeman, Eloise Stanton, Clifford C Sheckter

**Affiliations:** Stanford University, Stanford, California; Wake Forest University, Winston Salem, North Carolina; Keck School of Medicine of USC, Los Angeles, CA; Santa Clara Valley Medical Center, San Jose, CA; Stanford University, Stanford, California; Wake Forest University, Winston Salem, North Carolina; Keck School of Medicine of USC, Los Angeles, CA; Santa Clara Valley Medical Center, San Jose, CA; Stanford University, Stanford, California; Wake Forest University, Winston Salem, North Carolina; Keck School of Medicine of USC, Los Angeles, CA; Santa Clara Valley Medical Center, San Jose, CA; Stanford University, Stanford, California; Wake Forest University, Winston Salem, North Carolina; Keck School of Medicine of USC, Los Angeles, CA; Santa Clara Valley Medical Center, San Jose, CA; Stanford University, Stanford, California; Wake Forest University, Winston Salem, North Carolina; Keck School of Medicine of USC, Los Angeles, CA; Santa Clara Valley Medical Center, San Jose, CA

## Abstract

**Introduction:**

Discharge to acute rehabilitation is the gold standard following major burn injury. However, numerous barriers to rehabilitation exist, including race, payor type, and burn center type. It remains unclear whether the organizational structure of burn centers is associated with differences in discharge to rehabilitation. This study aims to investigate associations between race and burn center type in relation to the rate of discharge to acute rehabilitation.

**Methods:**

Using the California Health Care Access and Information from 2009-2019, all inpatient encounters at verified and non-verified burn centers were identified using International Classification of Diseases 9/10 and Diagnosis Related Group codes. The primary outcome of interest was proportion of patients discharged to acute rehabilitation. Key covariates included race, burn center safety-net status, American Burn Association (ABA) verification status, and American College Surgeons (ACS) Level 1 trauma center designation. Adjusted covariates included burn total body surface area (TBSA), gender, and age. Logistic regression modeling was performed, and multiple testing was accounted for using Bonferroni adjustment.

**Results:**

There were 27,496 encounters of which 0.8% (228) were discharged to inpatient rehabilitation. By race/ethnicity, the proportion of admission to inpatient rehabilitation was 0.9% for White, 0.6% for Black, 0.7% for Hispanic, and 1% for Asian. After adjusting for TBSA and age, notable predictors for discharge to inpatient rehabilitation included Medi-Cal (Medicaid) as payor (OR 1.24-2.22, p=0.005) compared to commercial insurance, trauma center status (OR 1.37-3.16, p=0.005), ABA verification status (OR 1.16-2.69, p=0.040), and safety-net facility (OR1.12-2.01, p=0.030). On univariate testing Black race and Hispanic ethnicity were associated with decreased odds of discharge to inpatient rehabilitation (p=0.033). This relationship was erased when these patients were treated at verified burn centers and/or burn centers with safety-net status.

**Conclusions:**

Discharge to inpatient rehabilitation remains variable between burn centers and races. Notably, verified burn centers with safety-net designation had more patients that discharged to inpatient rehabilitation adjusting for burn severity and payor. Governmental insurance was favorable for discharge to acute rehabilitation.

**Applicability of Research to Practice:**

The organization of burn centers and triage of burn patients may consider the benefits of verification and safety-net designation as it relates to discharge to inpatient rehabilitation.

